# The promoting effects of GPR176 expression on proliferation, chemoresistance, lipogenesis and invasion of oesophageal cancer

**DOI:** 10.1007/s00432-023-05256-2

**Published:** 2023-08-16

**Authors:** Wen-jing Yun, Jun Li, Nan-chang Yin, Cong-yu Zhang, Zheng-guo Cui, Li Zhang, Hua-chuan Zheng

**Affiliations:** 1 Department of Oncology, The Affiliated Hospital of Chengde Medical University, Chengde, 067000 China; 2 Department of Thoracic Surgery, Shandong Provincial Hospital, Jinan, 250021 China; 3 Department of Thoracic Surgery, The First Affiliated Hospital of Jinzhou Medical University, Jinzhou, 121001 China; 4 Cancer Center, The First Affiliated Hospital of Jinzhou Medical University, Jinzhou, 121001 China; 5 Department of Environmental Health, University of Fukui School of Medical Sciences, Fukui, 910-1193 Japan

**Keywords:** Oesophageal cancer, GPR176, Aggressiveness, Prognosis, Targeted therapy

## Abstract

**Purpose:**

As a member of the G-protein-coupled receptor 1 family, the G-protein-coupled receptor 176 (GPR176) gene encodes a glycosylated protein made up of 515 amino acids. The current study was performed to evaluate the impact of GPR176 on the clinicopathology and prognosis of oesophageal cancer, as well as uncover its molecular mechanisms.

**Methods:**

Bioinformatics and clinical tissue samples were used to detect the expression and clinicopathological significance of GPR176 in oesophageal cancer. The expression, proliferation, migration and invasion, apoptosis and lipid droplet formation of GPR176 gene in oesophageal cancer were performed as phenotypic readouts.

**Results:**

Here, RT-PCR and bioinformatic analyses revealed that GPR176 mRNA expression was significantly higher in oesophageal cancer than in normal mucosa (p < 0.05). GPR176 mRNA expression was associated with low weight and BMI, low T stage, low N and clinicopathological stage, low histological grade and favourable clinical outcome of oesophageal cancer (p < 0.05). The differential genes of GPR176 mRNA were involved in protein digestion and absorption, extracellular matrix constituent, endoplasmic reticulum lumen, among others (p < 0.05). GPR176-related genes were classified as being involved in oxidoreductase activity, actin and myosin complexes, lipid localisation and transport, among others (p < 0.05). GPR176 knockdown suppressed proliferation, anti-apoptotic and anti-pyroptotic properties, migration, invasion, chemoresistance and lipid droplet formation in oesophageal cancer cells (p < 0.05), while ACC1 and ACLY overexpression reversed the inhibitory effects of GPR176 silencing on lipid droplet formation and chemoresistance.

**Conclusion:**

These findings indicated that upregulated expression of GPR176 might be involved in oesophageal carcinogenesis and subsequent progression, aggressiveness, and induced chemoresistance by ACC1- and ACLY-mediated lipogenesis and lipid droplet assembly.

## Introduction

The incidence of oesophageal cancer (OC) is increasing with changes in the environment and the human diet. Adenocarcinoma (Ad) and Squamous cell carcinoma (Sq) are the two most common types of oesophageal cancer, which are caused by distinct genetic alterations that occur in various parts of the oesophagus. Squamous cell carcinoma (Sq) is more common than adenocarcinoma (Ad). Its risk factors include older age, male sex, smoking, alcohol consumption, exposure to polycyclic aromatic hydrocarbons, gastroesophageal reflux disease, dysplasia and tooth loss. Nonsteroidal anti-inflammatory drugs, vitamins, vegetables, green tea and fruit intake can prevent oesophageal carcinogenesis. Taking into account the patient’s health and the stage of the tumor, endoscopic removal may be used to treat early OC tumors, while advanced tumors may necessitate chemotherapy, chemo-radiotherapy, surgical resection or a combination of these approaches (Sawicki et al. 2021; Saadati et al. 2021). Despite advances in the handling and care of those with OC, the overall result of treatment remains highly unsatisfactory. To boost the potency of therapeutic interventions, there is a need to find biomarkers and molecular targets.

Integral membrane proteins known as human G-protein-coupled receptors (GPCRs) feature seven-membrane-spanning helices and can be divided into five distinct families: glutamate, rhodopsin, adhesion, frizzled and secretin (Fredriksson et al. 2003). GPCRs can attach to their natural ligands and interact with Gα, thus initiating the regulation of essential effectors and the production of secondary messengers, which consequently lead to the activation of downstream signal pathways (Flock et al. 2017). Structurally, GPCRs are characterised by active and inactive conformations, and they are capable of spontaneously shifting between these two forms at a baseline activity level that is not dependent on an agonist. When ligands, from cyclic AMP to peptides and large proteins, come into contact with GPCRs, these receptors undergo a change in conformation that leads to the activation of heterotrimeric G proteins, allowing the transmission of extracellular signals intracellularly. GPCR kinases and β-arrestins are known as the key regulators of GPCR signalling, as they collaborate to control GPCR desensitisation and trafficking through ubiquitination, phosphorylation and β-arrestins (Bar-Shavi et al. 2016). As shown in a review by Fredriksson et al. (2003), more than 800 GPCR sequences and 342 distinct functional sequences have been identified, making them ideal targets for drug treatment of diseases (Hauser et al. 2017).

GPR176, also known as HB-954 and Gm1012, is a member of the G-protein-coupled receptor 1 family. Located on human chromosome 15q14-q15.1, the GPR176 gene encodes a 515-aa protein (Hata et al. 1995). Wang et al. (2020) found that endogenous GPR176 is N-glycosylated at four conserved asparagine residues in the N-terminal region. Additionally, missense variations in the conserved N-glycosylation sites of human GPR176 (rs1473415441 and rs761894953) were shown to affect N-glycosylation and reduce protein expression and cAMP-repressive activity in cells. Kakarala and Jamil (2014) hypothesised that GPR176 might have the ability to interact with free fatty acids as a ligand. Moreover, findings reported by Schultz et al. (2018) indicated that anacardic acid could increase GPR176 expression in both MCF-7 and MDA-MB-231 breast cancer cells, as determined by RNA-seq. Furthermore, Forest et al. (2021) reported that GPR176 expression was higher in high-grade tumors of diffuse malignant epithelioid mesothelioma in TCGA analysis. Our study found that the expression of GPR176 protein was associated with an older age, a smaller tumor size and the non-luminal B subtype of breast cancer. Additionally, GPR176 knockdown was observed to reduce the proliferation, glucose catabolism, anti-apoptotic and anti-pyroptotic properties, migration, invasion and epithelial-mesenchymal transition (EMT) of breast cancer cells (Yun et al. 2023). Against this background, the current study was performed to evaluate the impact of GPR176 on the clinicopathology and prognosis of oesophageal cancer, as well as uncover its molecular mechanisms.

## Materials and methods

### Cell culture and transfection

An oesophageal squamous cancer cell line (KYSE-150) was obtained from the Cell Bank of the Chinese Academy of Sciences, Shanghai, China. The cells were kept in an atmosphere of 5% CO_2_, with temperature of 37 °C and humidity of 60%, in RPMI-1640 medium with 10% fetal bovine serum (FBS), 100 units/mL penicillin and 100 μg/mL streptomycin. The KYSE-150 cells were transfected with shGPR176 using Lipofectamine 3000 (Thermo Fisher Scientific). The target of shGPR176 is 5ʹ-GAGAGTGAGGCCAAGTACA-3ʹ. The cells were subjected to treatments with two substances: 5-fluorouracil (5-FU), a thymidylate synthetase inhibitor; and Taxol, an inhibitor of mitosis.

### Proliferation assay

The number of viable cells was determined using Cell Counting Kit-8 (CELLCOOK, Guangzhou, China). Initially, 2.0 × 10^3^ cells/well were seeded in a 96-well plate and allowed to adhere. Subsequently, 10 μL of CCK-8 solution was added to each well of the plate and incubated for 3 h in an incubator. The absorbance was then measured at 450 nm.

### Apoptosis assay by flow cytometry

Flow cytometry was conducted using 7-aminoactinomycin (7-AAD) and phycoerythrin (PE)-labeled annexin V (manufactured by Keygen, China) to ascertain phosphatidylserine externalisation, which serves as an indicator of early apoptosis, in accordance with the manufacturer’s recommendations.

### Wound healing assay

At a density of 1.0 × 10^6^ cells/well, six-well culture plates were seeded and the cell monolayer was allowed to reach confluence. A pipette tip was then used to scrape the monolayer and the area was washed three times with PBS before culturing in FBS-free medium. Pictures of the scratch area were taken at 24 h and the scratch length was measured using Image J software.

### Cell migration and invasion assays

For migration assays, 2.5 × 10^5^ cells were resuspended in serum-free RPMI-1640 and seeded in the control-membrane insert on the top of the chamber (BD Biosciences). The lower compartment of the chamber contained 10% FBS as a chemo-attractant. After incubation for 24 h, cells on the membrane were scrubbed, washed with PBS, fixed in 100% methanol and stained with Giemsa dye. For invasion assays, the procedures were the same as above apart from the use of a Matrigel-coated insert (BD Biosciences).

### Nile red staining

Following the fusion of cells in the six-well plate to a certain degree, the culture medium was discarded, the cells were washed with PBS two to three times and then fixed with methanol. Subsequently, 1 ml of Nile red dye solution (1 mg/ml) was added to each well for 2 min, and the nucleus was stained with DAPI (4,6-diamino-2-phenylindole). Images were acquired and analysed using Image J software.

### Patients

Paraffin-embedded and frozen oesophageal cancerous tissue and matched normal mucosa samples were collected from The First Affiliated Hospital of Jinzhou Medical University (China) between 2010 and 2021 for the construction of a tissue microarray and for protein extraction. Tissue and cDNA microarrays of oesophageal cancerous tissue and normal mucosa were purchased from Shanghai Outdo Biotech (Shanghai) and used for immunohistochemistry and RT-PCR, respectively. The University Ethical Committee approved the research protocol after the patients provided written informed consent for the use of tumor tissue for clinical research. No chemotherapy, radiotherapy or adjuvant therapy was administered to any of the patients before the surgery.

### qRT-PCR

Total RNA of fresh specimens was isolated using RNeasy Mini Kit (74104; Qiagen, Germany), quantified, and then cDNA was reverse-transcribed using M-MLV and random primers (Takara, Japan). NCBI’s primer-BLAST was employed to design real-time primers based on sequences from GenBank. The primers were as follows: GAPDH: forward 5′-CAATGACCCCTTCATTGACC-3′, reverse 5′-TGGAAGATGGTGATGGGATT-3′; and GPR176: forward 5′-TCCCTGCTATTGCTTTGGAC-3′, reverse: 5′-TACTGCAAACACAGGGACAC-3′. Amplification of the cDNA was achieved using the Cobas z480 real-time system (Roche), with iTaq™ Universal SYBR^®^ Green Supermix (172-5121; Bio-Rad, USA) used as the reagent and GAPDH functioning as an internal control.

### Western blotting

Fresh samples were lysed with RIPA lysis buffer and the proteins were quantified using the BCA Protein Assay Kit (Beyotime, China). Equal volumes of the proteins were then separated by 10% SDS-PAGE and transferred onto PVDF membranes. To prevent nonspecific antigen sites, 5% skim milk was applied for 1 h and the primary antibody was then incubated overnight at 4 °C. The membranes that had been washed three times were then incubated for 2 h with a 1:5000 dilution of anti-rabbit antibody with horseradish peroxidase (#7074S; CST, USA). C300 (Azure Biosystems, USA) was used to capture protein bands, which were then detected and measured using Image J software (v1.8.0) with the assistance of Western Bright™ ECL western blotting detection kit (K-12045-D50; Advansta, USA).

### Tissue microarray (TMA)

For the tissue microarray, the pathological specimens were fixed in 4% paraformaldehyde, followed by dehydration with alcohol, and then dealcoholisation with xylene, before embedding in paraffin. Sections of 4 μm thickness were sliced from the paraffin blocks, and hematoxylin-and-eosin staining was employed for histological analysis. Through microscopic examination, areas of normal tissue adjacent to solid tumors were identified and tissue cores were taken from the paraffin blocks and placed in pathological blocks, which were then sliced into 4-μm-thick sections.

### Immunohistochemistry (IHC)

The slides were deparaffinised and rehydrated three times in succession and antigen retrieval was then conducted in a microwave oven for a period of 20 min. A 30-min application of 3% hydrogen peroxide (H_2_O_2_) and 5% bovine serum albumin (BSA) was used to suppress endogenous peroxidase activity and avoid non-specific binding. Subsequently, slides were exposed to a rabbit anti-GPR176 antibody (1:60, ab122605; Abcam, USA) for 3 h at room temperature. After being washed with PBS three times, the slides were exposed to a polyclonal swine anti-rabbit antibody conjugated to HRP (1:200, P0399; DAKO, Japan) for 2 h at room temperature. DAB was employed to identify the precise binding sites, which were then stained with haematoxylin. Subsequently, the slides were dehydrated, cleared, mounted and observed under a microscope (Nikon Corporation, Japan). The IHC assessment was completed using the aforementioned method (Yun et al. 2023).

### Bioinformatics analysis

The expression of the GPR176 gene was analysed with the xiantao platform (https://www.xiantaozi.com/) and/or UALCAN database (http://ualcan.path.uab.edu). The prognostic significance of GPR176 was explored using Kaplan–Meier Plotter (http://kmplot.com/). The differentially expressed genes were used to construct a PPI network and selected as important hub genes using Cytoscape. GO + KEGG and GSEA analyses were conducted on these genes to construct signal pathways.

### Statistical analysis

SPSS 23.0 was used to conduct chi-squared test and Cox analysis. Spearman’s correlation analysis, Student’s t-test and log-rank test were used to compare the different rates, the difference between two groups and conduct survival analysis. P < 0.05 was considered to indicate statistical significance.

## Results

### Clinicopathological significance of GPR176 mRNA expression in oesophageal cancer

**Fig. 1:**
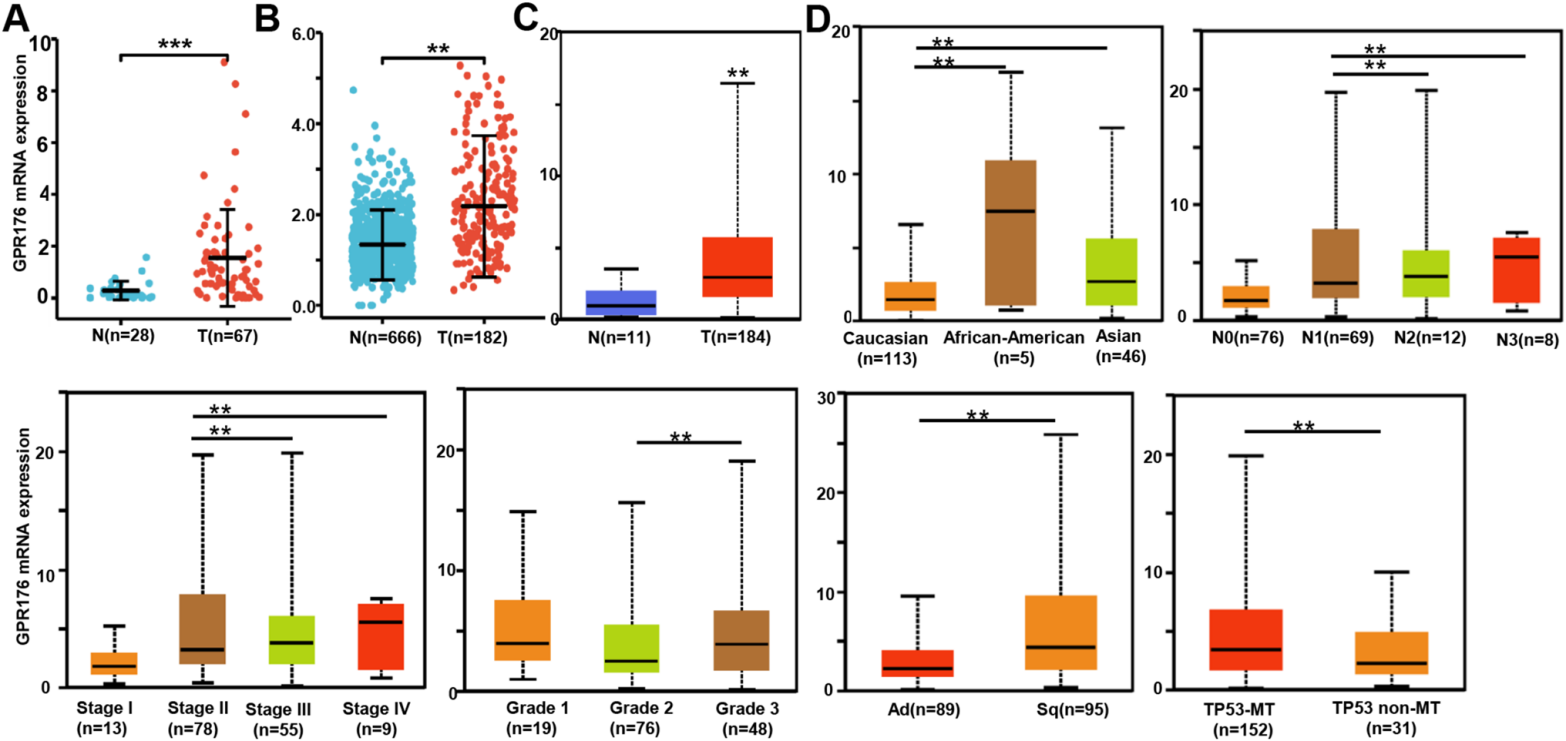
The clinicopathological significance of GPR176 mRNA expression according to bioinformatic analysis. A comparison of GPR176 mRNA expression was performed between oesophageal normal and cancer tissues by real-time PCR (**A**), and using Xiantao (**B**) and UALCAN databases (**C**). The association of such expression with clinicopathological features of oesophageal cancer was also analysed using the UALCAN database (**D**). *N* normal, *T* tumor, *Ad* adenocarcinoma, *Sq* squamous cell carcinoma

First, we performed real-time RT-PCR and found that GPR176 mRNA expression was higher in oesophageal cancer than in normal tissue (Fig. 1A, p < 0.05), in line with the data from both xiantao (Fig. 1B, p < 0.05) and UALCAN datasets (Fig. 1C, p < 0.05). According to UALCAN, such expression was lower in Caucasian cancer patients than in African-American and Asian ones, in N1 than in N2 and N3, in Stage II than in Stage III and IV cancer patients, in G2 than in G3 cancer patients, in adenocarcinoma than in squamous cell carcinoma patients, and in cancer patients without mutant p53 than in those with mutant p53 (Fig. 1D, p < 0.05). As shown in Table 1, GPR176 expression was gradually increased from distal to proximal sites of oesophageal cancer (p < 0.05). It was also found to be associated with low weight and BMI, low T staging and clinicopathological state, low histological grade, and favourable clinical outcome of oesophageal cancer (p < 0.05).

**Table 1:** The relationship between GPR176 mRNA expression and oesophageal cancer using the xiantao database

Characteristic	Variables	Low expression	High expression	p
Gender, n (%)	Female	13 (8%)	10 (6.2%)	0.653
	Male	68 (42%)	71 (43.8%)	
Age (years), n (%)	≦ 60	39 (24.1%)	44 (27.2%)	0.530
	> 60	42 (25.9%)	37 (22.8%)	
Tumor location, n (%)	Distal	65 (40.4%)	48 (29.8%)	**0.011**
	Mid	15 (9.3%)	27 (16.8%)	
	Proximal	1 (0.6%)	5 (3.1%)	
Weight, n (%)	≦ 70	29 (18.1%)	47 (29.4%)	**0.004**
	> 70	52 (32.5%)	32 (20%)	
BMI, n (%)	≦ 25	32 (20.9%)	52 (34%)	** < 0.001**
	> 25	47 (30.7%)	22 (14.4%)	
T stage, n (%)	T1	19 (13.1%)	8 (5.5%)	**0.008**
	T2	18 (12.4%)	19 (13.1%)	
	T3	30 (20.7%)	47 (32.4%)	
	T4	0 (0%)	4 (2.8%)	
N stage, n (%)	N0	28 (19.4%)	38 (26.4%)	0.444
	N1	32 (22.2%)	31 (21.5%)	
	N2	6 (4.2%)	3 (2.1%)	
	N3	2 (1.4%)	4 (2.8%)	
M stage, n (%)	M0	57 (44.2%)	64 (49.6%)	0.723
	M1	3 (2.3%)	5 (3.9%)	
Pathologic stage, n (%)	Stage I	10 (7%)	6 (4.2%)	0.410
	Stage II	32 (22.5%)	37 (26.1%)	
	Stage III	19 (13.4%)	30 (21.1%)	
	Stage IV	3 (2.1%)	5 (3.5%)	
Histologic grade, n (%)	G1	3 (2.4%)	13 (10.3%)	**0.035**
	G2	36 (28.6%)	30 (23.8%)	
	G3	20 (15.9%)	24 (19%)	
Primary outcome, n (%)	PD	9 (9.6%)	1 (1.1%)	**0.015**
	SD	5 (5.3%)	2 (2.1%)	
	PR	2 (2.1%)	1 (1.1%)	
	CR	32 (34%)	42 (44.7%)	

*BMI* body mass index, *PD* progressive disease, *SD* stable disease, *PR* partial response, *CR* complete response

**Fig. 2:**
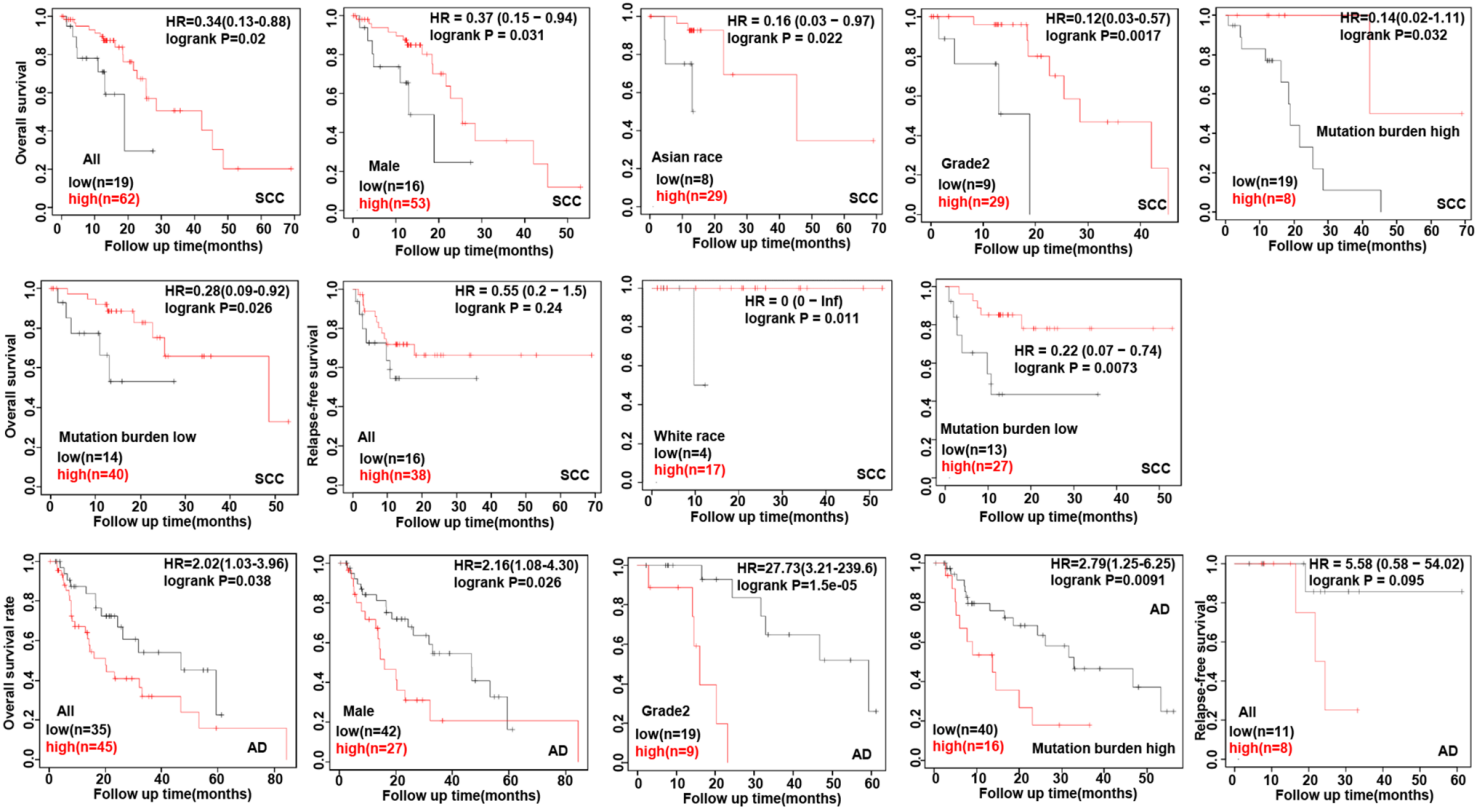
The prognostic significance of GPR176 mRNA expression in oesophageal cancer according to Kaplan–Meier Plotter. *SCC* squamous cell carcinoma, *AD* adenocarcinoma

In terms of the KM Plotter database (Fig. 2), GPR176 mRNA was positively associated with a higher overall survival (OS) rate in all, male and Asian oesophageal squamous carcinoma patients (p < 0.05), and in cancer patients of Grade 2 with a high or low mutation burden. The same results were found for relapse-free survival (RFS) for all and Caucasian squamous carcinoma patients or those with a low mutation burden (p < 0.05). As for oesophageal adenocarcinoma, GPR176 mRNA expression was negatively related to the OS of all and male cancer patients, and those at Grade 2 and with a high mutation burden (p < 0.05). The same was found for the RFS of all cancer patients (p < 0.05).

### Genes and signal pathways related to GPR176 in oesophageal cancer

**Fig. 3:**
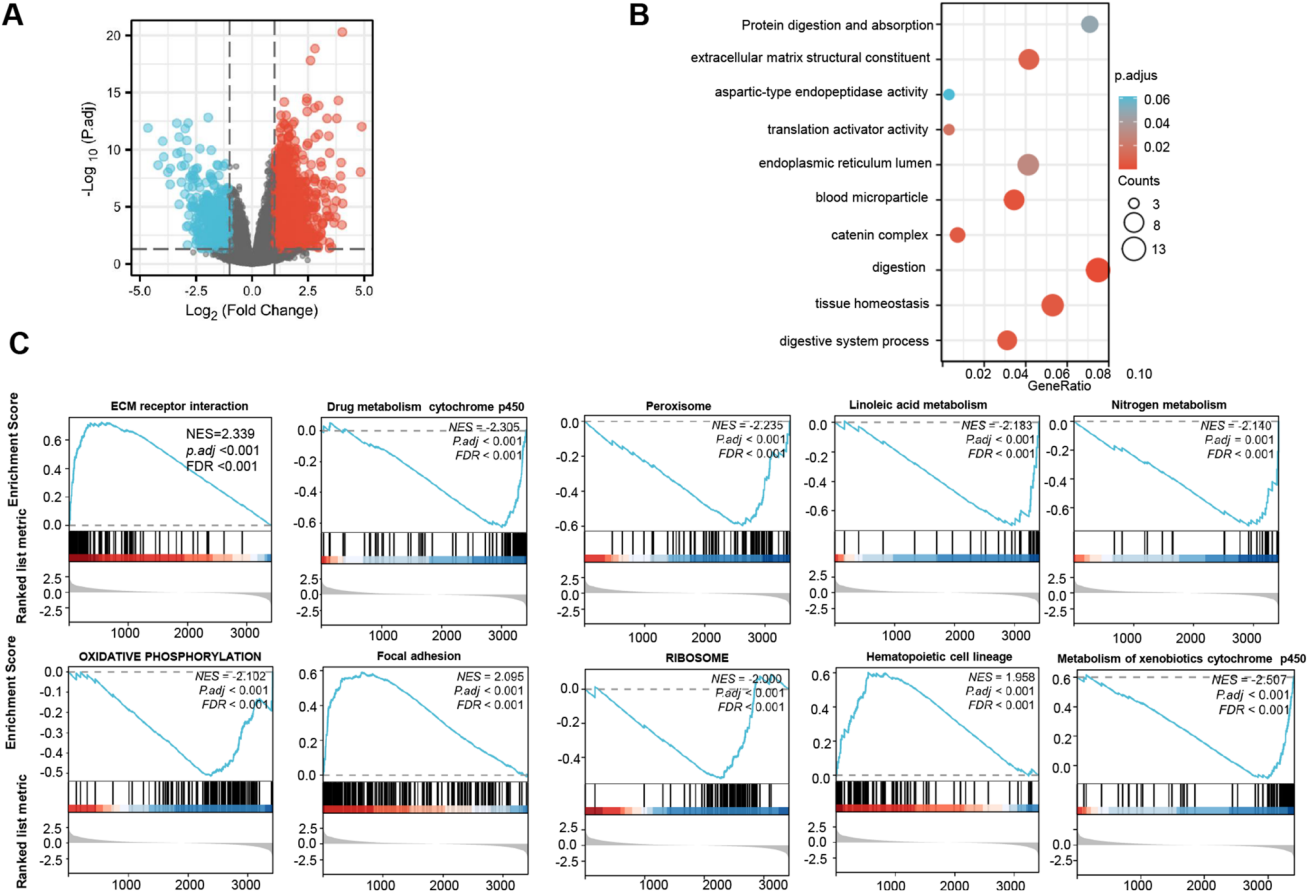
The genes and related signal pathways differentially expressed between oesophageal cancer cases with low and high GPR176 expression. A volcano map of the genes differentially expressed between low and high GPR176-expressing oesophageal cancer cases (**A**). These genes were subjected to signal pathway analysis using GSEA (**B**)

**Fig. 4:**
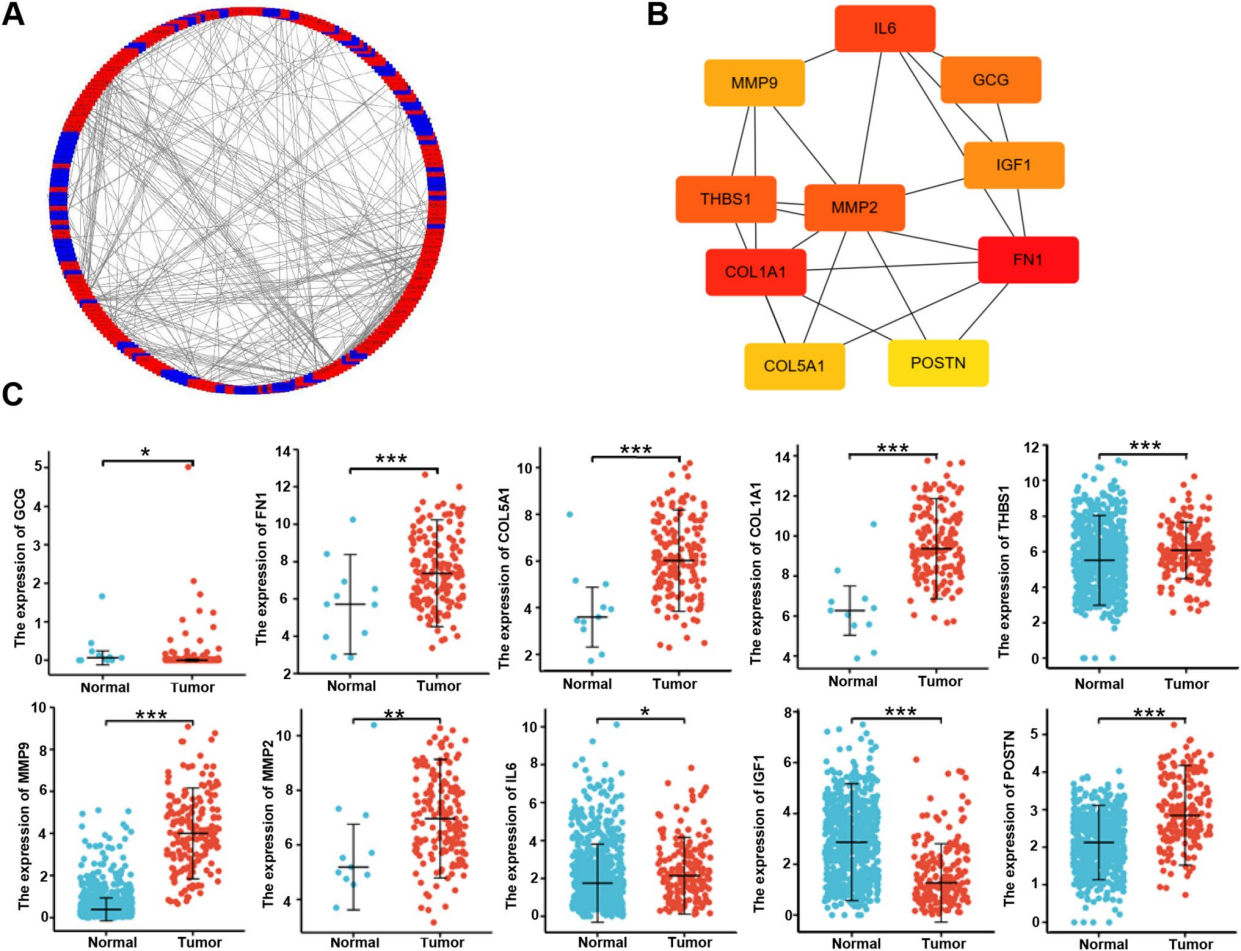
The hub genes of GPR176 in oesophageal cancer. Both String and Cytoscape were employed to screen the hub genes of GPR176 in oesophageal cancer (**A**). The hotspot hub genes were selected (**B**) and compared between oesophageal cancer and normal tissues (**C**)

In the xiantao platform, we conducted an analysis of genes differentially expressed between the low and high GPR176 mRNA expression groups in oesophageal cancer, and constructed a volcano map as shown in Fig. 3A. KEGG analysis revealed that the top signal pathways included protein digestion and absorption, extracellular matrix constituent, catenin complex, endoplasmic reticulum lumen and endopeptidase activity (Fig. 3B, p < 0.05). GSEA showed that the top signal pathways included ECM receptor interaction, peroxisome, focal adhesion, metabolism of drug and nitrogen (Fig. 3C, p < 0.05). STRING was employed to recognise the PPI pairs (Fig. 4A), while Cytoscape was used to determine the top 10 nodes ordered by degree (Fig. 4B). Results from the xiantao database revealed that GCG and IGF1 had lower expression levels in oesophageal cancer than in normal tissues (Fig. 4C, p < 0.05), whereas FBN1, COL5A1, COL1A1, THBS1, MMP9, MMP2, IL6 and POSTN showed the opposite trend (Fig. 4C, p < 0.05).

**Fig. 5:**
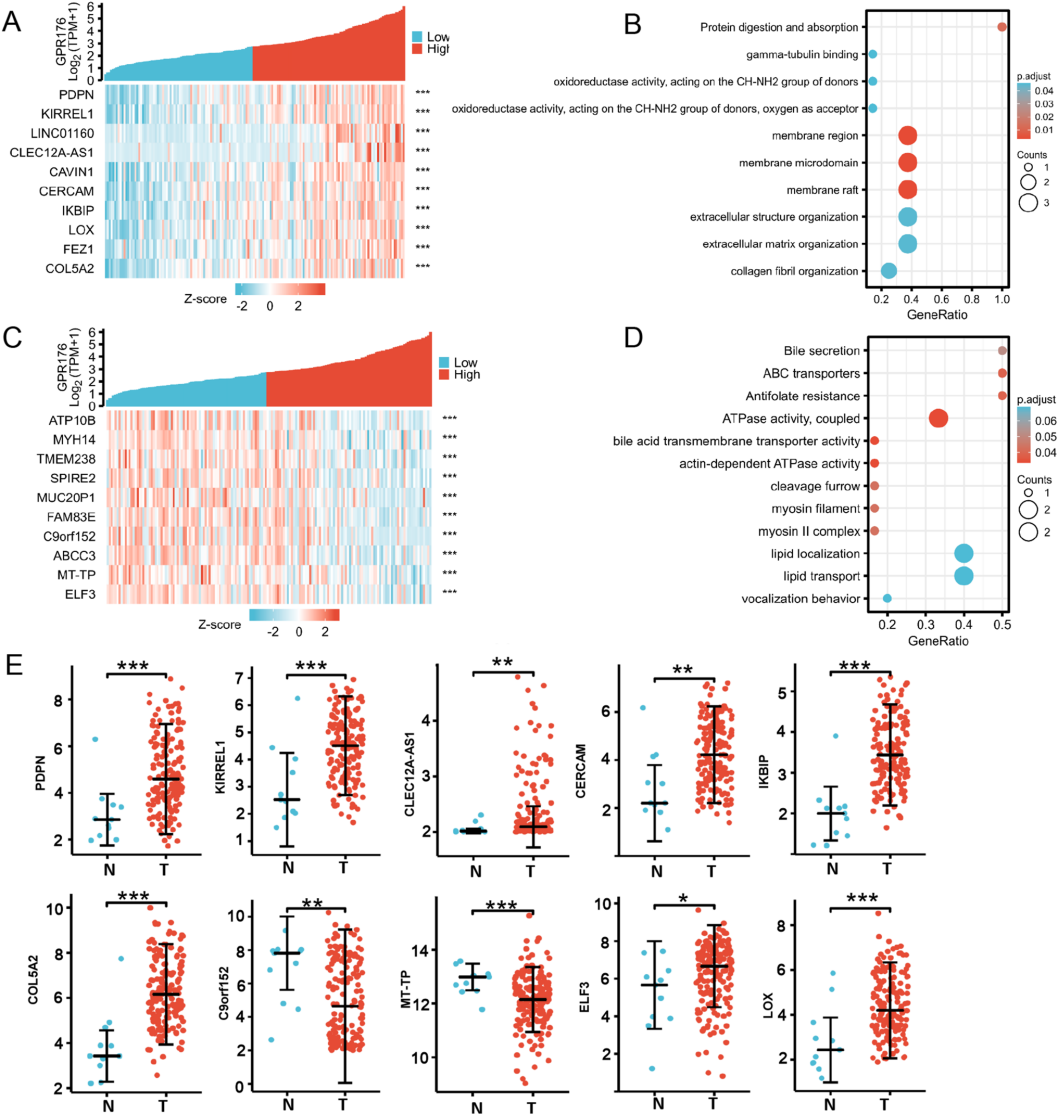
The GPR176-related genes and signal pathways in oesophageal cancer. The genes positively associated with GPR176 were screened (**A**) and classified into signal pathways using the xiantao database (**B**). The genes negatively associated with GPR176 were screened (**C**) and classified into signal pathways using the xiantao database (**D**). The expression profile of these genes were studied using the xiantao platform (**E**)

The genes whose expression was positively correlated with GPR176 in oesophageal cancer according to the xiantao database are shown in Fig. 5A (p < 0.05). These genes were shown to be involved in oxidoreductase activity, membrane region and raft, and extracellular organisation, among others (Fig. 5B, p < 0.05). The genes whose expression was negatively correlated with GPR176 in oesophageal cancer are shown in Fig. 5C (p < 0.05). These were found to be involved in bile and bile acid metabolism, actin and myosin complexes, and lipid localisation and transport (Fig. 5D, p < 0.05). The top GPR176-correlated genes (PDPH, KIREL1, CLEC12A-AS1, CERCAM, IKBIP, LOX, COL5A2 and ELF3) were more highly expressed in oesophageal cancer than in normal tissue (Fig. 5E, p < 0.05), but the converse was true for C9orf152 and MT-TP (Fig. 5E, p < 0.05).

### Clinicopathological and prognostic significance of GPR176 protein expression in oesophageal cancer

**Fig. 6:**
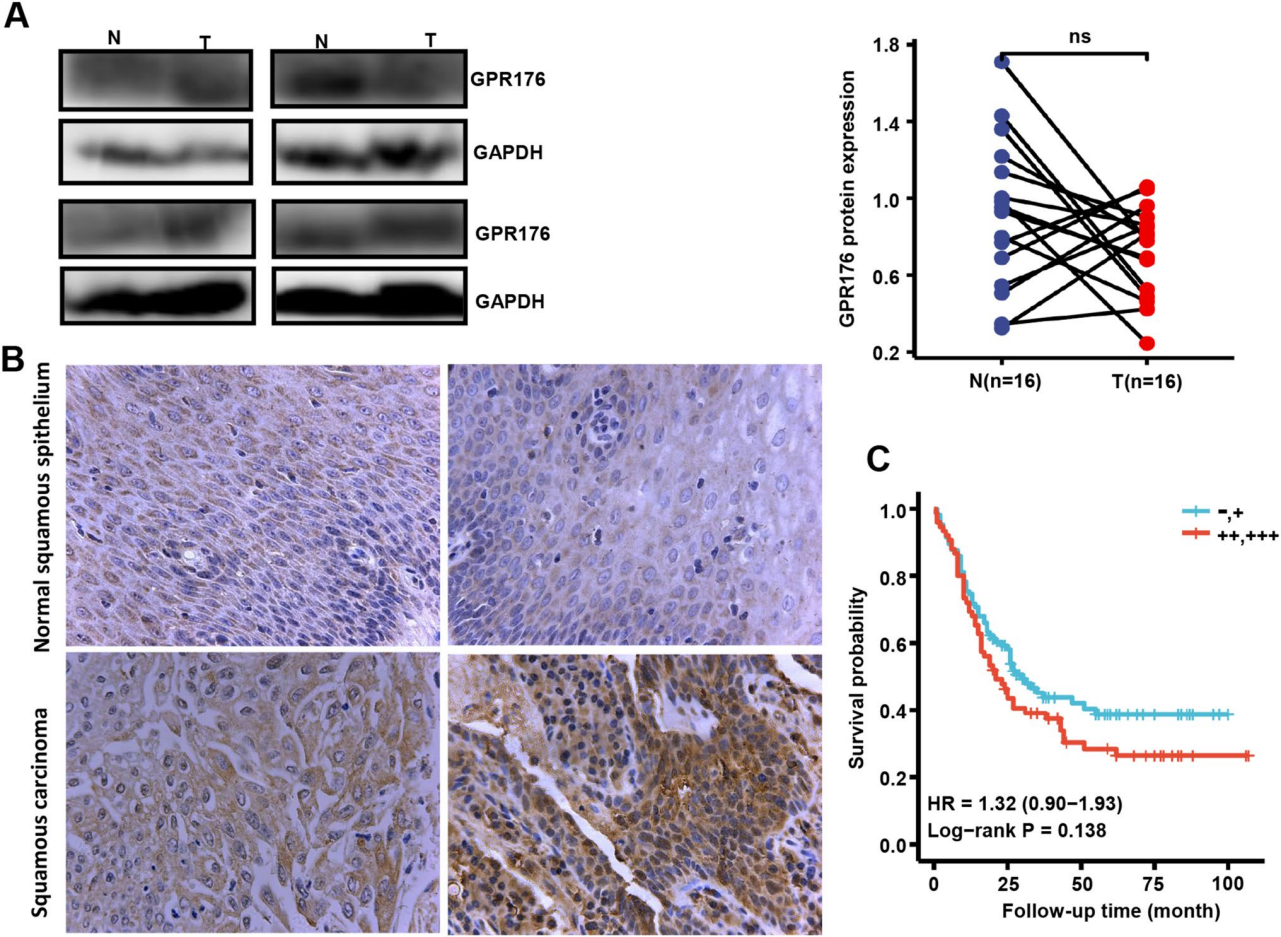
The clinicopathological significance of GPR176 protein expression in oesophageal cancer. Western blotting was used to determine the level of GPR176 protein in oesophageal cancer (**A**). Densitometric analysis showed no difference in GPR176 expression between oesophageal cancer and normal tissues (**A**, p > 0.05). Immunohistochemically, there was positivity for GPR176 protein expression in oesophageal squamous cancer and epithelial and cancer cells (**B**). Kaplan–Meier curves and log-rank test were used to clarify the prognostic significance of GPR176 protein expression (**C**). *N* normal, *T* tumor, *ns* not significant, *HR* hazard ratio

No difference in GPR176 expression was observed between oesophageal cancer and matched normal tissues when densitometric analysis of western blotting was conducted (Fig. 6A, p > 0.05). Immunohistochemically, there was positivity for the expression of GPR176 protein in oesophageal squamous cancer and epithelial cells (Fig. 6B). No remarkable disparity in survival was detected between those with low and high expression of GPR176 (Fig. 6C). As summarised in Table 2, the rates of positivity for GPR176 expression were 76.9% (226/294) and 79.4% (259/326) in oesophageal normal mucosa and oesophageal cancer, respectively. Considering the frequency and density, GPR176 expression was positively correlated with high histological grade of oesophageal cancer, but was not associated with sex, age, T stage, N stage or AJCC staging of oesophageal cancer (Table 3, p < 0.05). Univariate analysis showed that sex, positive lymph node, T stage, N stage and AJCC stage were positively correlated with unfavourable overall survival of oesophageal cancer patients (Table 4, p < 0.05). Multivariate analysis showed that sex and AJCC stage were independent factors affecting the survival of oesophageal cancer patients (Table 4, p < 0.05).

**Table 2:** GPR176 expression in oesophageal cancer

Groups	n	GPR176 expression				
		−	+	++	+++	PR (%)
Normal tissue	294	68	151	54	21	76.9
Oesophageal cancer	326	67	160	61	38	79.4

*PR* positive rate

**Table 3:** The relationship between GPR176 protein expression and clinicopathological characteristics of oesophageal cancer by immunohistochemistry

Clinicopathological features	n	GPR176 expression				PR (%)	ρ	p value
		−	+	++	+++			
*Sex*							− 0.038	0.498
Female	46	8	23	8	7	82.6		
Male	276	58	136	52	30	79.0		
*Age (years)*							0.086	0.125
< 65	189	40	99	35	15	78.8		
≥ 65	132	26	60	24	22	80.3		
*Histological* grade							− 0.128	**0.046**
I–II	199	37	94	41	27	81.4		
III	43	13	21	5	4	69.8		
*T staging*							− 0.026	0.651
T1	21	2	14	4	1	90.5		
T2	58	10	28	15	5	82.8		
T3	224	51	111	36	26	77.2		
T4	13	3	4	3	3	76.9		
*N staging*							− 0.015	0.794
N0	139	25	75	30	9	82.0		
N1	95	14	46	17	18	85.3		
N2	70	22	30	9	9	82.9		
N3	13	3	5	4	1	76.9		
*AJCC staging*							− 0.008	0.888
I	18	3	12	3	0	83.3		
II	128	20	70	26	12	84.4		
III	162	40	70	29	23	75.3		
IV	5	1	3	0	1	80.0		

*PR* positive rate

**Table 4:** Survival analysis of oesophageal cancer patients by immunohistochemistry

Variables	Univariate analysis			Multivariate analysis		
	β	HR (95% CI)	p value	β	HR (95% CI)	p value
Sex (male vs. female)	0.598	1.819 (1.245–2.656)	**0.002**	0.611	1.842 (1.008–3.365)	**0.047**
Age (≥ 65 vs. < 65 years)	− 0.17	0.874 (0.642–1.117)	0.24	− 0.2	0.821 (0.550–1.224)	0.333
Positive lymph nodes (≥ 2 vs. < 2)	0.629	1.875 (1.411–2.491)	** < 0.001**	− 0.02	0.982 (0.506–1.906)	0.956
T staging (T1–2 vs. T3–4)	0.715	2.044 (1.339–3.120)	**0.001**	− 0.11	0.899 (0.486–1.663)	0.735
N staging (N0–1 vs. N2–3)	0.807	2.241 (1.643–3.057)	** < 0.001**	0.305	1.356 (0.707–2.600)	0.359
Histological grade (I–II vs. III)	0.018	1.019 (0.738–1.406)	0.911	0.196	1.216 (0.779–1.897)	0.389
AJCC staging (I–II vs. III–IV)	0.784	2.190 (1.635–2.934)	** < 0.001**	0.914	2.495 (1.372–4.537)	**0.003**
GPR176 expression (−, + vs. ++, +++)	0.278	1.321 (0.910–1.918)	0.144	− 0.36	0.699 (0.407–1.202)	0.195

*HR* hazard ratio, *CI* confidence interval

### Effects of GPR176 expression on the aggressiveness of oesophageal cancer cells

**Fig. 7:**
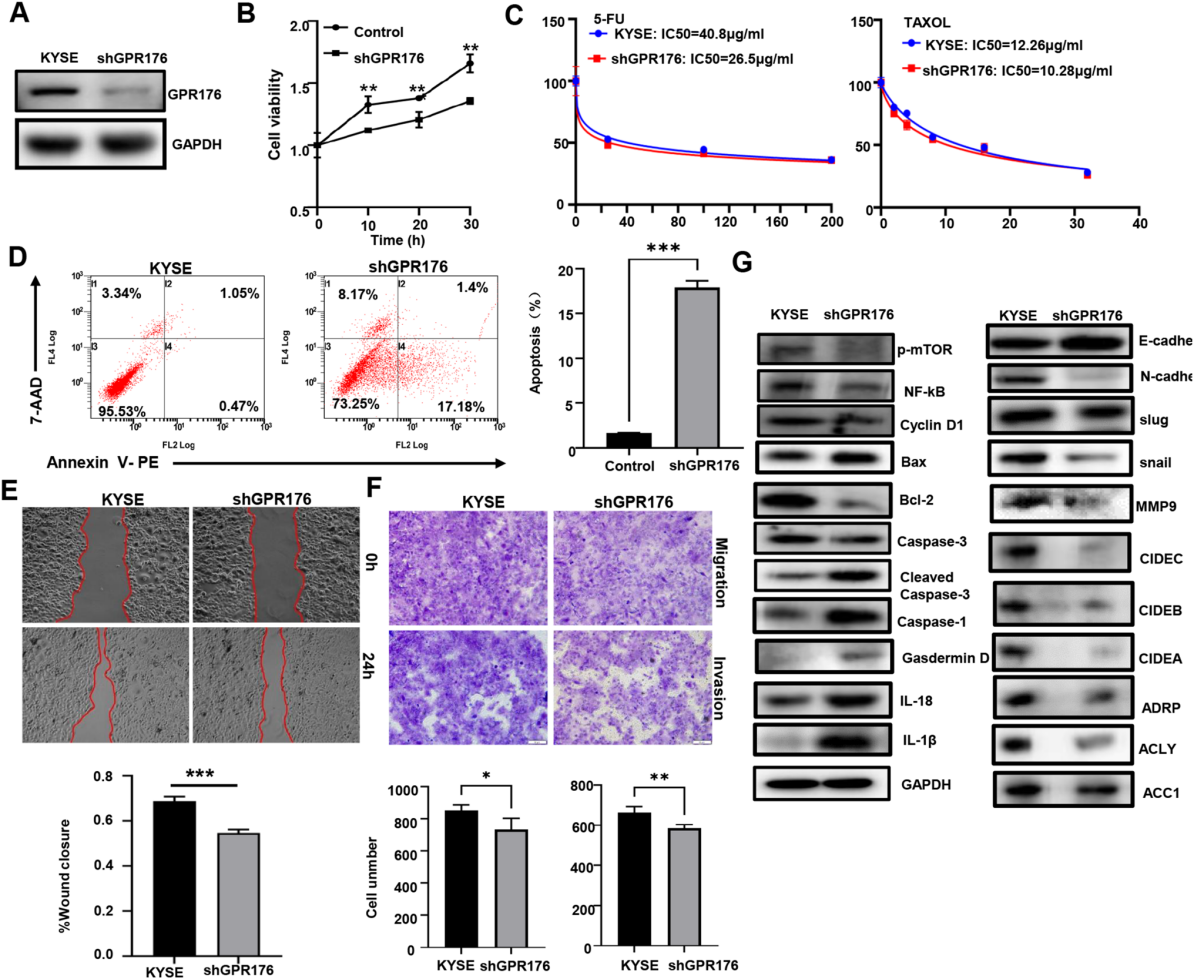
The effects of GPR176 expression on the phenotypes and molecular mechanisms of oesophageal cancer cells. After transfection of shGPR176, GPR176 expression became weaker in KYSE-150 cells, as determined by western blotting (**A**). The transfectants were subjected to functional assays of proliferation, chemoresistance, apoptosis, migration and invasion using CCK-8 (**B**, **C**), Annexin V/7-AAD staining (**D**), wound healing assay (**E**) and transwell assay (**F**), respectively. The proteins associated with these phenotypes were screened by western blotting (**G**). KYSE, KYSE-150 cells; *p < 0.05; **p < 0.01; ***p < 0.001

After transfection with shGPR176, KYSE-150 cells showed low expression of GPR176 protein, as revealed by western blotting (Fig. 7A). The rate of growth of shGPR176 transfectants was also slower than that of control cells (Fig. 7B, p < 0.05). In addition, GPR176 silencing caused chemosensitivity to 5-FU and Taxol (Fig. 7C). Moreover, KYSE-150 cells exhibited a high rate of apoptosis after shGPR176 transfection (Fig. 7D, p < 0.05). Compared with the levels of control cells, KYSE-150 cells with low GPR176 expression also exhibited decreased migration and invasion capacities, as revealed by wound healing assays (Fig. 7E, p < 0.05) and Transwell assays (Fig. 7F, p < 0.05). Furthermore, as indicated in Fig. 7G, GPR176 knockdown decreased the levels of expression of p-mTOR, NF-κB, Cyclin D1, Bcl-2, Slug, Snail, MMP9, CIDEC, CIDEB, CIDEA, ADRP, ACC1 and ACLY, but increased the levels of expression of Bax, Cleaved caspase-3, IL-18, IL-1β, Caspase-1, and E-cadherin in KYSE-150 cells.

**Fig. 8:**
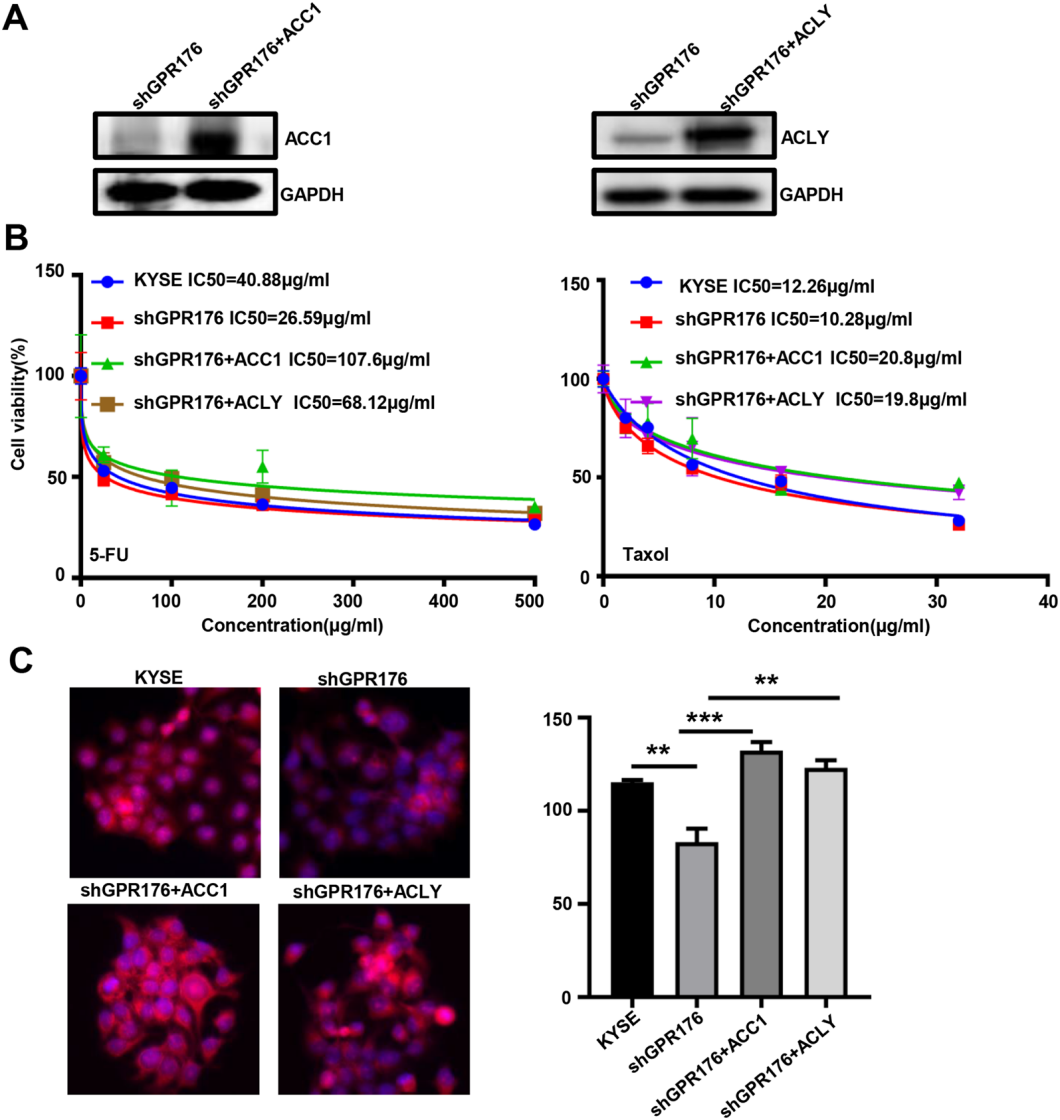
The effects of ACC1 and ACLY on GPR176-mediated chemoresistance and lipogenesis. Either ACC1 and ACLY was overexpressed in shGPR176 transfectants of KYSE-150 cells, as confirmed by western blotting (**A**). After 5-FU or taxol treatment, KYSE-150 cells and transfectants were analysed using CCK-8 (**B**) and Nile red staining (**C**). KYSE, KYSE-150 cells; **p < 0.01; ***p < 0.001

To confirm the effects of ACC1 and ACLY on GPR176-mediated chemoresistance and lipogenesis, we overexpressed ACC1 and ACLY, as confirmed by western blotting (Fig. 8A). Either ACC1 or ACLY increased the chemoresistance of shGPR176 transfectants of KYSE-150 cells against 5-FU and Taxol and lipid droplet formation, as revealed by CCK-8 (Fig. 8B) and Nile red staining (Fig. 8C, p < 0.05), respectively.

## Discussion

When a ligand binds to a GPR, it undergoes a conformational change, thus enabling it to act as a guanosine exchange factor. This is achieved through the replacement of GDP on the G protein with GTP, which leads to the separation of the α subunit from the β and γ subunits. This process activates the G protein’s α subunit, allowing it to bind to GTP and consequently commencing the subsequent step of signalling. The particular activated signalling pathway, namely, the cAMP or phosphatidylinositol pathway, is determined by the type of α subunit (GαS, GαI/O, GαQ/11 and Gα12/13). Sequencing of the human genome has revealed nearly a thousand G-protein-coupled receptor genes. The receptors encoded by these genes can be classified into six types: Classes A (rhodopsin-like receptors), B (secretin receptor family), C (metabolic glutamate receptors), D (fungal mating pheromone receptors), E (cyclic adenylate receptors) and F (frizzled/smoothened family) (Fong et al. 1988; Matsuoka et al. 1988; Martin et al. 2015).

In ovarian cancer, we found that GPR176 mRNA expression positively correlated with older age, clinicopathological staging and tumor residual status (Yang et al. 2023), while GPR176 expression at both mRNA and protein levels was associated with low T staging and good PAM50 classification of breast cancer (Yun et al. 2023). Ni et al. (2023) first found that GPR176 was negatively associated with a low clinical stage, favourable prognosis and chemosensitivity, and involved in the stromal remodeling of gastric adenocarcinoma. In line with the findings on gastric cancer (Ni et al. 2023), we found that GPR176 mRNA expression was upregulated in oesophageal cancer, and positively correlated with T and N clinicopathological staging, and dedifferentiation of oesophageal cancers. This indicated that GPR176 mRNA overexpression might be involved in the carcinogenesis and subsequent progression of oesophageal cancer. However, no difference in GPR176 protein expression was identified between oesophageal cancer and normal tissues, while such expression was positively associated with histological grading, in line with the findings for GPR176 mRNA. This suggests that GPR176 is involved in the differentiation of oesophageal cancer at both mRNA and protein levels. Notably, GPR176 mRNA was positively correlated with favourable clinical outcome, suggesting its value for predicting treatment efficacy. Moreover, GPR176 knockdown has been found to impede the proliferation, glucose catabolism, anti-apoptotic and anti-pyroptotic properties, migration, invasion and epithelial-mesenchymal transition of breast cancer cells (Yun et al. 2023), which is in agreement with results obtained from ovarian cancer cells (Yang et al. 2023). We hypothesised that GPR176 could be a factor contributing to the development and progression of oesophageal cancer, as it appears to worsen the aggressiveness of gastric cancer cells.

Studies on ovarian cancer have demonstrated that GPR176 mRNA was associated with lower overall, progression-free and post-progression survival rates, regardless of the stratification of clinical parameters (Yang et al. 2023). It has also been revealed that GPR176 mRNA expression is associated with a favourable outcome for the relapse-free survival of all and ER-positive cancer patients, and the overall survival of PR-positive cases (Yun et al. 2023). However, such expression has a negative association with the overall survival or post-progression survival of cancer patients with lymph node involvement, or the distant-metastasis-free survival of ER-positive cases (Yang et al. 2023). In the present study, GPR176 mRNA was positively associated with higher OS in oesophageal SQ, while having the opposite association for OS and RFS of oesophageal AD. The level of GPR176 mRNA was also higher in SQ than in AD. The prognostic significance of GPR176 mRNA was possibly dependent on the histological subtype, even the distinct expression of GPR176 between SQ and AD. GPR176 protein was not correlated with the prognosis of oesophageal cancer patients, but sex, T staging, N staging and TNM staging were shown to be prognostic risk factors. Among these, TNM staging was an independent risk factor for the prognosis of oesophageal cancer patients. Taking these findings together, GPR176 might be available as a biological marker to predict the outcome of oesophageal cancer patients.

GPR176 has been found to activate the cAMP/PKA pathway in colorectal cancer, and its transmembrane helix 3-intracellular loop 2 domain has been observed to recruit GNAS, thereby amplifying the intracellular GPR176 signal. Additionally, this GPR176-GNAS complex has been shown to inhibit mitophagy through the cAMP/PKA/BNIP3L pathway. Reportedly, GPR176-related genes were shown to be involved in receptor-ligand interaction, RNA maturation, cell mobility and membrane structure in breast cancer (Yun et al. 2023), and to contribute to focal adhesion, ECM-receptor interaction, ribosome, oxidative phosphorylation, actin skeleton, cytokine-cytokine receptor interaction, gap junction and cell adhesion molecules in ovarian cancer (Yang et al. 2023). We also found that GPR176-related signal pathways included extracellular matrix constituent, catenin complex, endoplasmic reticulum lumen, endopeptidase activity, ECM-receptor interaction, peroxisome, focal adhesion, membrane region and raft, extracellular organisation, actin and myosin complexes, and lipid localisation and transport. Taking these findings together, we hypothesise that GPR176 might strengthen the aggressiveness of oesophageal cancer cells by affecting cell adhesion and mobility, membrane and lipid rafts.

Overactivation of the PI3K/Akt/mTOR pathway has frequently been observed to be associated with proliferation and anti-apoptotic properties in a range of different cancers (Sanaei et al. 2022). It has been found that Bcl-2, when it interacts with Bax on the mitochondrial membrane, can prevent Bax from opening the mitochondrial voltage-dependent anion channel, thus hindering apoptosis (He et al. 2022). In oesophageal cancer cells, GPR176 silencing was shown to ameliorate proliferation and induce apoptosis by either inactivating PI3K/Akt/mTOR or decreasing Bcl-2/Bax. Pyroptosis is a newly revealed form of inflammatory programmed necrosis that is mediated by Gasdermin D and Caspase-1, resulting in cell death (Arakelian et al. 2022). Meanwhile, Slug and Snail have been found to promote EMT in association with E-cadherin overexpression and N-cadherin underexpression (Fedele et al. 2022). Through our research, we determined that GPR176 knockdown could stimulate pyroptosis and inhibit the EMT of oesophageal cancer by reducing the levels of Slug and Snail. Meanwhile, MMPs are well known to break the extracellular matrix and promote metastasis (Wieczorek et al. 2015). In this regard, it was shown that GPR176 silencing reduced the expression of MMP9, which explained GPR176’s effects of promoting the invasion and metastasis of gastric cancer cells.

Upon GPR176 knockdown, KYSE-150 cells developed chemosensitivity to 5-FU and Taxol and weakened lipid droplet formation. Reportedly, chemoresistance of colorectal cancer cells was produced by LPCAT2-mediated lipid droplet formation (Cotte et al. 2018), which was also aided by prothymosin α (Jin et al. 2021), and metastasis-associated in colon cancer (Duan et al. 2017) through SREBP-1- and FASN-mediated lipogenesis respectively. Crucial enzymes for de novo fatty acid synthesis are ACC1 and ACLY, which are closely linked to chemoresistance (Sur et al. 2019). In the liver and peritoneal tissues, lipid droplet assembly is mediated by ADRP and CIDEs (Fan et al. 2020; Kasano-Camones et al. 2020). GPR176-mediated lipid droplet formation might be closely linked to the expression of ADRP, CIDEA, CIDEB and CIDEC. GPR176-induced lipogenesis might be remarkably associated with the expression of ACC1 and ACLY. Moreover, GPR176-mediated lipogenesis might account for the GPR176-induced chemoresistance against 5-FU and Taxol because ACC1 and ACLY overexpression might reverse the inhibitory effects of GPR1176 knockdown on lipid droplet formation and chemoresistance. Taking these findings together, we hypothesised that GPR176 may have a role in chemoresistance via both de novo lipogenesis and lipid droplet assembly.

In summary, GPR176 is believed to be involved in the pathogenesis and subsequent progression of oesophageal cancer by promoting proliferation, anti-apoptotic and anti-pyroptotic properties, migration, invasion and EMT of oesophageal cancer cells. GPR176 might induce chemoresistance by ACC1- and ACLY-mediated lipogenesis and lipid droplet assembly in oesophageal cancer cells.

## Data Availability

There is no data needed to be deposited. The datasets generated and/or analyzed during the current study are available from the corresponding author on reasonable request.
